# The health benefits of bicycling to school among adolescents in China: A propensity score matching study

**DOI:** 10.3389/fpubh.2023.1049254

**Published:** 2023-04-04

**Authors:** Pengxiang Ding, Chang Ding, Suwei Feng

**Affiliations:** ^1^School of Public Economics and Administration, Shanghai University of Finance and Economics, Shanghai, China; ^2^School of Science and Information, Qingdao Agricultural University, Qingdao, China

**Keywords:** bicycling to school, adolescents, subjective health, objective health, sickness absence

## Abstract

**Background:**

A large number of high-income countries are now promoting active commuting to school as an opportunity for adolescents to increase physical activity (PA) and improve their health. Few studies have examined the multiple benefits of active travel to school among adolescents in developing countries, especially in China. Hence, this study aims to estimate the effects of bicycling to school on adolescents’ subjective health, physical health, and sickness absence.

**Methods:**

Self-reported and cross-sectional data from 6,353 school-aged children (12–19 years old) in the 2014–2015 China Education Panel Survey (CEPS2014-2015) were used. The independent variable was a binary, self-reported indicator of whether children bicycled to school. The dependent variables included subjective health (self-reported health, mental stress), physical health (BMI, kidney disease, lung disease, heart disease, brain disease, upper limb fracture, lower limb fracture, and sickness frequency), and sickness absence. Propensity score matching (PSM) was used to estimate the causal effects of bicycling to school on adolescents’ health.

**Results:**

Bicycling to school positively affects both subjective and physical health. Those students who biked to school were associated with a higher self-rated health status, a healthier weight, a lower level of mental stress, and a lower risk of developing brain diseases. No significant relation is found between bicycling and sickness frequency, and sickness absence. Moreover, we separately compared the bicycling group with the walking group and the non-active travel group. There is still evidence that cycling is beneficial for students. Compared with walking to school, cycling to school resulted in a higher self-rated health score and a lower mental stress score. Physically, students who bicycled to school were less likely to be absent from school and suffer from kidney and brain diseases than students who walked to school. However, we do not find a significant difference in health outcomes from cycling compared to non-active modes of transportation. Further, differentiation of the health effects of bicycling across living areas shows that health effects are more pronounced for those living in edge and rural areas.

**Conclusion:**

These findings provide evidence of the value of promoting bicycling to school in improving various adolescents’ health outcomes in transitional China.

## 1. Introduction

Insufficient physical activity among school-age adolescents is one of the most common problems worldwide. In China, 84.3% of adolescents were insufficiently physically active (1). Physical inactivity is linked to many chronic diseases, such as heart disease (2), kidney disease (3), and colon cancer (4). Moreover, according to the latest statistics released by the National Health Commission (2018), about 30 million Chinese children under 17 also suffer from various mood disorders and problems requiring comprehensive interventions (5). The prevalence highlighted the need for increased physical activity opportunities in adolescents to improve their physical and mental health.

Active commuting, defined as walking or bicycling to school, can easily incorporate PA into school-age children’s daily life. Many researchers have examined a broad range of health benefits associated with active travel (often combining walking and bicycling). Studies have shown that active commuting is associated with better perceived physical health (6–9), increased levels of physical activity (10), as well as lower body mass indexes (11). Some studies further examined the role of active travel on mental health. Active commuting is associated with more positive emotions (12–14) and lower stress (15–18) than commuting by passive modes. Furthermore, active commuting has been found to reduce sickness absence (19, 20).

Some studies, however, have highlighted the different contributions of bicycling and walking to the health benefits (21), and a crude grouping could conceal some health benefits behind the choice of travel mode. Walking is the physical activity that is more prevalent among the population than bicycling and sufficient evidence for promoting walking to/from school (22). However, taken as a whole, there was less evidence supporting the health benefit of bicycling compared to walking. Further, cycling is more energy-intensive than walking (8). It was reported that vigorous activity has a greater health impact than moderate physical activity (23). Therefore, cycling to school may have a greater potential for increasing various health indicators than walking (24, 25). More research is needed to understand the relationship between biking to school and adolescents’ health.

A large number of high-income countries are now promoting active commuting to school as an opportunity to increase adolescents’ PA and improve their health (26–29). However, such efforts remain unclear in developing countries like China. To the authors’ knowledge, although China has the world’s largest school system, only one empirical study on active commuting to school among national representative adolescents has been conducted (11, 12, 30), and empirical evidence in previous literature in the western context may not apply directly to China’s unique situation. First, with economic growth and greater use of automobile transportation in China, active commuting to school among young people has decreased from 84% in 1997 to 55.8% in 2010 (11, 12, 30). Second, the policy of school merging was implemented in rural areas from 2000 to 2010. After the adjustment to the school layout, the commuting distance for rural students was extended from 1.6 to 4.0 km (31), and long commuting time could have opposing effects on health (32, 33), the impact of bicycle to school on adolescents’ health may be canceled out. Third, compared to developed countries, China’s rapid urbanization has led to severe environmental problems (34), which may reduce the positive effects of bike commuting. Given these unique contexts, it is urgent to examine the effects of bicycling on Chinese adolescents’ health so that effective policy can be developed.

We identify four gaps in the literature that motivate this research. First, previous studies show that a significant correlation exists between active travel to school and health, mostly based on surveys of a single health indicator; few papers comprehensively included various health outcomes in one analysis. Second, the conclusion reached by previous studies that a significant correlation exists between active commuting and health has been mostly combined with bicycling and walking together. There was less evidence, especially on the health benefit of bicycling. Third, previous research on child commute well-being has been dominated by studies from the U.S., Canada, and Europe, while few studies have paid specific attention to the development contexts of adolescents. Fourth, the association between commutes and health is complex and difficult to confirm. Most previous studies have examined the association through regression analysis, while few have explored the causal association using econometric techniques.

To fill these knowledge gaps, this study takes a more comprehensive approach to explore the effect of bicycling on adolescents’ health: (a) We investigate whether bicycling to school improves children’s health across several domains (subjective health, physical health, and sickness absence) rather than just one domain such as mental status or physical activity. (b) Using data from a nationally representative survey called the China Education Panel Survey; this paper examines the relationship between adolescent bicycle commuting and their health in China, the world’s largest developing country. (c) Unlike previous studies that combined bicycle and walking trips as active commuting studies, specifically, this study compares the health benefits of bicycling with those of non-bicycling, walking, and other modes of passive commuting. (d) We use an econometric technique known as propensity score matching (PSM) to investigate causal effects rather than correlations. The methodology is described in the following section.

## 2. Method

### 2.1. Data

The data for this paper is drawn from the China Education Panel Survey (CEPS), the first nationally representative longitudinal survey of junior-high students conducted by the National Survey Research Center at the Renmin University of China. The data have been used in numerous studies, confirming their validity (35–40). The survey includes extensive information on students’ socio-demographics, school travel modes, health, school management, and teacher qualities. The baseline survey is a random sample (applied a multistage sampling method with probabilities proportional to size) of approximately 20,000 students in 438 classrooms of 112 schools in 29 county-level units in mainland China in 2013–2014 and 2014–2015. Only the 2014–2015 CEPS collected information on student school travel time and mode, so we use the 2014–2015 CEPS. There are four types of questionnaires (students, parents, teachers, and school administrators) in the survey. This study uses student questionnaires that are completed by students in the classroom, and all variables used are self-reported by the students. After excluding the sample of boarding students, the final sample includes 6,353 eighth-grade students,^1^ ranging in age from 12 to 19, with a mean age of 14.03 and a standard deviation of 0.79.

### 2.2. Outcomes variables

We examine adolescents’ health across different domains (Table 1), ranging from subjective health to sickness absence.

**Table 1:** Description of the variables.

Variables	Definition
**Dependent variable**	
Cycling to school	Cycling to school = 1
**Independent variables**	
Self-reported health	Very poor = 1 to very good = 5
Mental stress	Score scale from 1 to 50
BMI	Healthy weight = 1, overweight/obese = 0
Kidney disease	Yes = 1
Lung disease	Yes = 1
Heart disease	Yes = 1
Brain disease:	Yes = 1
Upper limb fracture	Yes = 1
Lower limb fracture	Yes = 1
Sickness frequency	Never = 1, seldom = 2, often = 3
Sickness absence	Days in sick during the last year
**Control variables**	
Gender	Male = 1
Only-child	Only-child = 1
Hukou	Rural = 1
Migrant children	Yes = 1
Physical activity	Min/week
Commute times	Number of minutes from home to school
Father’s education	Year
Mother’s education	Year
Family socioeconomic	Poor = 1 to very rich = 5
Residential district	Central area = 1, outskirts =2; rural =3
School rank	5-point Likert scale
Teacher’s education	The number of teachers has obtained a bachelor’s degree
School type	Public school = 1
County fixed effect	28 dummy variables

Subjective health. The widely used subjective health measure is self-rated health, which was measured by the question ‘Which one of the following best describes your general health condition at present’. Responses were five options: 1 = very poor, 2 = not very good, 3 = moderate, 4 = good, and 5 = very good. The second variable is mental stress, measured through a set of 10 questions derived from the extended version of the Patient Health Questionaire-9 (35). The question was ‘How often have you felt (1) depressed (2) unfocused (3) unhappy (4) boring (5) could not work hard (6) sadness (7) tension (8) worry (9) something wrong will happen (10) too energetic and inattentive in class in the past 7 days?’. And each question had 5 options: 1 = never, 2 = seldom, 3 = sometimes, 4 = often, and 5 = always. We summed up the 10 emotional indicators with a value range of 0–50 (Cronbach’s α coefficient = 0.912), and a higher score means worse mental health.

Physical health. The first measure of physical adolescents’ health is body mass index (BMI), which is estimated using the standard equation (weight [kg]/height [m]^2^). Age- and gender-specific BMI z-scores were calculated based on the guidelines “overweight and obesity criteria for school-age children and adolescents” published by the Chinese National Health Commission in 2018^2^, participants were categorized as healthy and overweight/obese (Healthy weight = 1, overweight/obese = 0). The second physical health measure is how often someone called in sick (cold, fever, cough, diarrhea) during the last year. Responses included never (1), seldom (2), often (3). The third measure is whether the adolescent has ever had one of the six serious illnesses (kidney, lung, heart, brain, upper limb fracture, lower limb fracture). Each question had two options: yes (1) and no (2).

Sickness absence. We also use adolescents’ sickness absence as a health outcome, and the assessment is based on a self-reported measure of the number of days absent due to sickness in the previous year.

### 2.3. Exposures variables

Students in CEPS 2014–2015 were asked about their school travel mode. The question was: ‘what mode do you usually use from home to school?’ Respondents choose one of the 12 options: walking, bicycle, motorbike, electric bicycle, city bus, coach, private car, train, boat, underground, or other commuting modes. We are transforming commuting mode into a binary variable which equals 1 for adolescents bicycling to school and 0 otherwise. Based on this survey question, we created three dummy variables. One is a dummy variable that indicates whether a participant commutes by bicycle, and it takes the value of 1 if they commute by bicycle, and 0 otherwise. The second dummy variable indicates whether the participant bicycles or walks to travel, with a value of 1 for bicycle commuters and 0 for walkers. Lastly, the third dummy variable indicates whether the participant commutes by bicycle or passively, taking the value of 1 for bicycle commuters and 0 for passive commuters.

### 2.4. Covariates

The control variables in this study were roughly divided into three types.

Individual-level variables include gender (male = 1), only-child (yes = 1), Hukou^3^ (Chinese household registration system, rural = 1), migrant status (yes = 1), commute duration, and physical activity. The commute duration was measured by the following question: How long does it usually take you to travel from home to school (minutes)? Physical activity was measured based on the student’s self-reported time spent exercising in a week (minutes). The question asked ‘The amount of time you spend on physical activity: [__] days per week, [__] minutes per day,’ and the total number of exercise hours per week was calculated by multiplying the number of exercise days per week by the number of exercise hours per day.

Family background variables include parental education, socioeconomic status, and residential district. A student’s parental education was measured by the number of years for which his or her parents had been educated. Family socioeconomic status was measured by asking the question ‘What do you think of your family’s current economic condition?.’ Response options were: 1 = very poor, 2 = not very good, 3 = moderate, 4 = rich, and 5 = very rich. Residential districts were classified into three categories (1 = central area, 2 = outskirts area, 3 = rural area).

At the school level, indicators include type (public school = 1), ranking, and teacher education. It was requested that school administrators report a school’s local ranking on a scale ranging from 1 (lowest) to 5 (highest). Teachers’ education level is determined by how many of them have a college degree.

Moreover, traffic conditions, geography, or weather conditions may also affect the health benefits of cycling. Therefore, we included county fixed effects in the model to control for the effects of unobservable environmental factors.

### 2.5. Statistical model

We use propensity score matching (PSM) to explore the causal effects. The approach is to construct a synthetic control group and compare the bicycling outcomes of this group to the treatment group (41). Our PSM analysis consists of three steps. First, we predict propensity scores for every student using a logit model controlling all the covariates as mentioned above (42). Second, every treated student is matched to a controlled child, ensuring the two are as alike as possible apart from the commuting mode. To estimate the impact of bicycling on child health, we matched bicycle commuters with non-bicycle commuters based on socioeconomic and demographic characteristics at the individual, household, and county levels. Also, using the same control variables, we matched bicycle commuters with passive commuters or walkers. We use radius matching to estimate the causal effects, then use the neighbor and kernel matching for the robustness check. Third, the average treatment effects of bicycling can be computed as:


$$\mathrm{ATT}=E\left(Health_{1i}-Health_{0i}|Bicyling_{i}\right)$$

$$=\;\;E\left(Health_{1i}|Bicyling_{i}=1\right)$$

$$-\;\;E\left(Health_{0i}|Bicyling_{i}=0\right).$$

ATT represents the average treatment effects of bicycling on health. $Bicyling_{i}$ is a binary variable, reflecting whether the student *i* bicycling to school. $Health_{1i}$ refers to the health outcomes of the bicyclist, and $Health_{0i}$ is the *i*’s health with other commute modes.

## 3. Results

### 3.1. Descriptive statistics

As shown in Table 2, 21% of Chinese adolescents bicycled to school. Regarding subjective health, students who biked to school had better self-rated health and less mental stress than those who commuted by other modes. In terms of physical health, there was little difference in BMI among those with different commute modes. Bicycle commuters were less likely to suffer from kidney, lung, heart, and brain disease but had a higher probability of upper and lower extremity fractures. Moreover, bicycle commuters reported fewer sick days than non-bicycle commuters. Significant differences were also found in the other characteristics of adolescents between the treatment and control groups. A sample selection bias may result from comparing the two groups directly before matching. This bias indicates that the difference in health between the two groups may not only result from changes in commuting patterns, but may also arise from individual or household characteristics. Our choice of PSM for causal inference was primarily motivated by this consideration.

**Table 2:** Descriptive statistics.

Variable	Bicycle commuters					Non-bicycle commuters				
	Mean	*N*	SD	Min	Max	Mean	*N*	SD	Min	Max
**Independent variables**										
Self-reported health	3.936	1,321	0.922	1	5	3.883	4,982	0.940	1	5
Mental stress	21.232	1,301	8.234	10	50	21.536	4,917	8.337	10	50
BMI	0.867	1,295	0.340	0	1	0.886	5,026	0.318	0	1
Sickness absence	1.738	1,304	7.089	0	123	1.881	4,908	10.605	0	365
Kidney disease	0.004	1,327	0.061	0	1	0.007	5,026	0.085	0	1
Lung disease	0.038	1,327	0.190	0	1	0.046	5,026	0.210	0	1
Heart disease	0.006	1,327	0.077	0	1	0.008	5,026	0.090	0	1
Brain disease:	0.004	1,327	0.061	0	1	0.008	5,026	0.088	0	1
Upper limb fracture	0.063	1,327	0.242	0	1	0.057	5,026	0.232	0	1
Lower limb fracture	0.033	1,327	0.179	0	1	0.029	5,026	0.169	0	1
Sickness frequency	1.938	1,310	0.469	1	3	0.010	2,623	0.097	1	3
**Control variables**										
Gender	0.641	1,303	0.480	0	1	0.486	4,960	0.500	0	1
Only child	0.487	1,310	0.500	0	1	0.558	4,959	0.497	0	1
Hukou	0.290	1,327	0.454	0	1	0.352	5,026	0.478	0	1
Ethnic	0.941	1,323	0.236	0	1	0.915	5,007	0.279	0	1
Migrant children	0.809	1,309	0.393	0	1	0.783	4,936	0.412	0	1
Commute time	15.597	1,327	10.550	1	155	17.973	5,026	15.307	0	180
Physical activity	51.421	1,295	51.529	0	700	47.024	4,921	44.770	0	999
Father’s education	10.951	1,284	3.322	0	19	11.159	4,845	3.209	0	19
Mother’s education	10.400	1,277	3.435	0	19	10.640	4,826	3.522	0	19
Family socioeconomic	2.853	1,282	0.533	1	5	2.893	4,838	0.565	1	5
Residential district	1.878	1,277	0.837	1	3	1.826	4,790	0.847	1	3
School rank	4.096	1,327	0.820	1	5	3.983	5,026	0.841	1	5
School type	0.976	1,327	0.153	0	1	0.975	5,026	0.156	0	1
Teacher’s education	79.163	1,279	43.251	0	195	84.064	4,881	41.733	0	195

### 3.2. Benchmark results

We used a logit model to estimate the likelihood of bicycling to school using all the covariates, and the estimated coefficients were saved as the propensity scores. As shown in Table 3, the estimated values confirm that the covariates significantly affect the likelihood of bicycling. Boys, non-only child, and rural area students are more likely to bicycle to school (all *p* < 0.001). The ethnic, commute time, and school-level factors are also related to the travel mode.

**Table 3:** Propensity score (cycling to school vs. non-bicycling).

Variable	Coef.	Std. Err.	*p*-value	95% CI
Gender	0.633	0.072	0.000	0.493, 0.774
Only child	−0.382	0.079	0.000	−0.537, −0.226
Hukou	−0.286	0.081	0.000	−0.445, −0.127
Ethnic	0.410	0.144	0.004	0.129, 0.692
Migrant children	0.212	0.094	0.024	0.028, 0.397
Commute time	−0.015	0.003	0.000	−0.021, −0.009
Physical activity	0.001	0.001	0.179	0.000, 0.002
Father’s education	0.005	0.015	0.759	−0.025, 0.034
Mother’s education	−0.016	0.014	0.260	−0.045, 0.012
Family socioeconomic status	−0.095	0.065	0.146	−0.222, 0.033
Residential district	−0.057	0.049	0.239	−0.152, 0.038
School rank	0.251	0.048	0.000	0.157, 0.344
School type	0.433	0.263	0.100	−0.082, 0.949
Teacher’s education	−0.004	0.001	0.000	−0.006, −0.002

**Figure 1:**
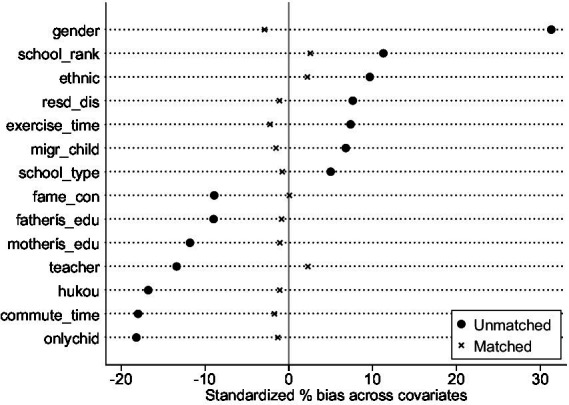
Propensity distributions of treated and control groups before and after matching.

Next, we conduct a balance check to ensure that samples are well-balanced. Table 4 and Figure 1 show that all variables’ standardized deviation (% bias) after matching was less than 5%. No systematic difference is found between the treatment and control groups, which means the parallel hypothesis^4^ is satisfied and the covariate balance is met.

**Table 4:** Parallel hypothesis in estimating the ATT of bicycling to school on the health.

Variable	Match	Treated	Control	%bias	%reduct	*t*	*p* > t
Gender	U	0.63	0.48	31.30		9.18	0.00
	M	0.63	0.64	−2.90	90.80	−0.69	0.49
Only child	U	0.48	0.57	−18.20		−5.40	0.00
	M	0.48	0.49	−1.30	92.80	−0.31	0.76
Hukou	U	0.29	0.37	−16.80		−4.88	0.00
	M	0.29	0.30	−1.10	93.60	−0.26	0.79
Ethnic	U	0.94	0.92	9.70		2.74	0.01
	M	0.94	0.93	2.20	77.00	0.56	0.58
Migrant children	U	0.82	0.79	6.80		1.99	0.05
	M	0.82	0.82	−1.50	77.60	−0.37	0.71
Time	U	15.49	17.86	−18.00		−4.91	0.00
	M	15.50	15.72	−1.70	90.40	−0.47	0.64
Exercise_time	U	50.63	47.20	7.40		2.21	0.03
	M	50.46	51.49	−2.20	69.80	−0.47	0.64
Father’s_edu	U	10.90	11.19	−9.00		−2.67	0.01
	M	10.90	10.93	−0.90	90.50	−0.20	0.84
Mother’s_edu	U	10.26	10.67	−11.80		−3.48	0.00
	M	10.26	10.30	−1.10	91.00	−0.25	0.80
Fame_con	U	2.85	2.90	−8.90		−2.61	0.01
	M	2.85	2.85	0.10	99.10	0.02	0.99
Resid_dis	U	1.89	1.82	7.60		2.26	0.02
	M	1.88	1.89	−1.10	85.50	−0.26	0.80
School_rank	U	4.10	4.00	11.30		3.32	0.00
	M	4.10	4.07	2.60	77.20	0.62	0.54
School_type	U	0.98	0.97	5.00		1.42	0.16
	M	0.98	0.98	−0.80	84.50	−0.20	0.84
Teacher_edu	U	79.55	85.18	−13.40		−4.01	0.00
	M	79.59	78.62	2.30	82.90	0.55	0.58

U, Unmatched; M, Matched.

Then, we used caliper matching for estimation, and the neighbor and kernel matching methods for robustness testing, and the results obtained by the three methods were generally consistent. As shown in Table 4, we found a significant positive effect of bicycling to school on students’ subjective health, including enhanced self-rated health (ATT = 0.06, SE = 0.04, *p* < 0.05), reduced mental stress (ATT = −0.73, SE = 0.33, *p* < 0.01). Regarding physical health, we found that bicycle commuters had a healthier weight (ATT = 0.03, SE = 0.15, *p* < 0.01) and were significantly less likely to have brain disorders (ATT = −0.01, SE = 0.00, *p* < 0.01). No significant relation is found among bicycling, sickness absence, and sickness frequency.

As shown in Table 5, we compared the bicycling group with both the walking and the non-active travel groups separately. There is still evidence that cycling generates beneficial outcomes for students. Cycling to school resulted in higher self-rated health (ATT = −0.12, SE = 0.05, *p* < 0.001) and a lower mental stress score (ATT = −0.82, SE = 0.37, *p* < 0.01) than walking. Physically, students who bicycled to school were less likely to be absent from school (ATT = −1.29, SE = 0.46, *p* < 0.001) and had kidney (ATT = −0.01, SE = 0.00, *p* < 0.001) and brain diseases (ATT = −0.01, SE = 0.00, *p* < 0.01). However, we did not find a significant difference in health outcomes between cycling and other non-active modes of transportation.

**Table 5:** Effects of bicycling on adolescent health (bicycling vs. walking; bicycling vs. non-active commute).

Variable	Bicycling vs. walking			Bicycling vs. non-active commute		
	ATT	SE	T-stat	ATT	SE	T-stat
Self-report health	0.12***	0.05	2.86	−0.02	0.08	−0.21
Mental stress	−0.82**	0.37	−2.23	0.00	0.68	0.01
BMI	0.02	0.01	1.32	0.02	0.03	0.88
Sickness absence	−1.29***	0.46	−2.83	0.06	0.75	0.07
Kidney disease	−0.01***	0.00	−2.89	−0.00	0.01	−0.26
Lung disease	−0.00	0.01	−0.23	−0.01	0.02	−0.81
Heart disease	−0.00	0.00	−0.44	0.00	0.01	0.24
Brain disease	−0.01**	0.00	−2.37	−0.00	0.01	−0.32
Upper limb fracture	−0.01	0.01	−0.68	0.00	0.02	0.21
Lower limb fracture	−0.01	0.01	−1.08	−0.01	0.01	−058
Sickness frequency	−0.01	0.02	−0.33	−0.04	0.03	−0.96

ATT, Average Treatment Effect on the Treated; SE, Standard Error; T-stat, T-statistic; **p < 0.01; ***p < 0.001.

There is a massive gap in economic development, transportation, and living environment between urban and rural areas in China. Therefore, this paper conducted a subsample regression based on students’ home locations. Table 6 shows that bicycling to school did not significantly affect students’ health in urban centers, while bicycle commuters living in the urban fringe (ATT = −1.72, SE = 0.77, *p* < 0.01), and rural areas (ATT = −1.33, SE = 0.65, *p* < 0.01) had lower mental stress. In addition, bicycle commuters in the urban fringe had a relatively lower probability of lower limb fractures (ATT = −0.03, SE = 0.01, *p* < 0.01). Moreover, bicycle commuters in rural areas are less likely to get kidney disease (ATT = −0.01, SE = 0.00, *p* < 0.05) and be absent on sick leave (ATT = −1.34, SE = 0.55, *p* < 0.01). In summary, we found that the health benefits of bicycle commuting were particularly pronounced for students in both urban edge and rural areas (Table 7).

**Table 6:** Effects of bicycling on adolescent health (bicycling vs. non-bicycling).

Variable	Radius matching			Neighbor matching			Kernel matching		
	ATT	SE	T-stat	ATT	SE	T-stat	ATT	SE	T-stat
Self-report health	0.06*	0.04	1.71	0.07*	0.04	1.81	0.07*	0.04	1.75
Mental stress	−0.73**	0.33	−2.18	−0.77**	0.37	−2.08	−0.72**	0.34	−2.16
BMI	0.03**	0.15	1.97	0.04***	0.02	−2.57	0.03	0.01	1.95
Sickness absence	−0.56	0.35	−1.61	−0.37	0.40	−0.93	−0.56	0.35	−1.59
Kidney disease	−0.01	0.00	−1.39	−0.00	0.00	−1.33	−0.01	0.00	−1.38
Lung disease	−0.01	0.01	−1.15	−0.01	0.01	−1.13	−0.01	0.01	−1.15
Heart disease	−0.00	0.00	−0.35	−0.00	0.00	−0.43	−0.00	0.00	−0.34
Brain disease	−0.01**	0.00	−2.26	−0.01**	0.00	−2.15	−0.01**	0.00	−2.29
Upper limb fracture	0.00	0.01	0.50	0.01	0.01	0.63	0.00	0.01	0.51
Lower limb fracture	−0.00	0.01	−0.07	−0.00	0.01	−0.35	−0.00	0.01	−0.13
Sickness frequency	−0.01	0.02	−0.61	−0.01	0.02	−0.51	−0.01	0.02	−0.64

ATT, Average Treatment Effect on the Treated; SE, Standard Error; T-stat, T-statistic; *p < 0.05; **p < 0.01; ***p < 0.001.

**Table 7:** Effects of bicycling on adolescent health in different regions.

Variable	Center			Fringe			Rural		
	ATT	SE	T-stat	ATT	SE	T-stat	ATT	SE	T-stat
Mental stress	0.25	0.66	−0.38	−1.72**	0.77	−2.24	−1.33**	0.65	−2.04
Self -report	0.07	0.07	0.90	0.04	0.09	0.46	0.07	0.08	0.92
BMI	0.00	0.02	0.21	−0.01	0.01	−0.58	−0.06	0.06	−1.04
Sickness frequency	0.03	0.04	0.72	0.03	0.04	0.92	0.01	0.04	0.34
Kidney disease	−0.00	−0.00	−0.63	−0.00	0.01	−0.19	−0.01*	0.00	−1.66
Lung disease	−0.01	0.01	−0.78	−0.00	0.01	−0.16	0.00	0.01	−0.02
Heart disease	−0.00	0.00	−0.21	−0.00	0.00	−0.31	0.00	0.00	0.46
Brain disease:	−0.00	0.00	−0.65	−0.01	0.01	−1.16	−0.00	0.01	−0.61
Upper limb fracture	0.02	0.02	1.31	−0.01	0.02	−0.41	0.00	0.01	0.17
Lower limb fracture	0.01	0.01	0.72	−0.03**	0.01	−2.17	0.00	0.01	0.13
Sickness absence	0.13	0.96	0.13	0.03	0.38	0.08	−1.34**	0.55	−2.44

ATT, Average Treatment Effect on the Treated; SE, Standard Error; T-stat, T-statistic; *p < 0.05; **p < 0.01.

## 4. Discussion

Based on data from the 2014–2015 China Education Panel Survey, this paper used propensity scores to design comparable treatment and control groups and then estimated the causal relationship between bicycling and students’ multidimensional health. The main results indicate that bicycling to school positively affects both subjective and physical health. No significant relation is found among bicycling, sickness frequency, and sickness absence.

One key finding of this study is that bicycling to school positively affected students’ subjective health compared with those who use other commute modes. First, bicycling can reduce students’ mental stress, possibly because the bicycle commute allows students to be in close contact with nature and the outside environment, stimulating positive emotions and reducing anxiety (43). Previous research has found that bicycle commuting effectively reduces mental anxiety in adults (15–17, 44, 45), and this paper shows that this effect is also present in adolescents. In a study involving Swedish children (10–15 years of age), Westman et al. (46) directly assessed the emotional state of the children upon arriving at school. Also, the results indicated that children were more likely to experience a positive feeling when they traveled by bicycle. With nearly 30 million Chinese adolescents suffering from mental anxiety, our study may prove that promoting bike commuting is an effective intervention to enhance Chinese adolescents’ mental health. Moreover, we also found that bicycling to school enhances students’ self-rated health. Compared to mental stress, self-rated health is a comprehensive indicator (47). This paper shows that mental stress and physical health may contribute to improving students’ self-rated health.

This study also identifies a positive association between bicycle commuting and students’ physical health. Notably, bicycle commuters were significantly less likely to suffer from brain diseases than the control group, and the probability of suffering from other diseases and frequency of illness were not affected. This finding further supports the mental health benefits of bicycling, as numerous studies have shown a correlation between mental stress and brain disease (48–50). Previous studies have shown that cycling for a prolonged period of time can improve adolescent BMI and reduce obesity (51, 52), we also found a positive relationship between bicycling to school and teenagers’ BMI. Thus, teenagers who engage in cycling as a form of extracurricular exercise can also effectively improve their body mass index (53).

Moreover, school attendance is vital for students’ academic performance and future development (54, 55), so we further investigated the relationship between bike commuting and students’ sick leave absences. Previous studies have found that active commuting reduces sick leave absenteeism in adults (19, 20, 56), but we did not find this effect in adolescents.

Also, this study compares bicycling trips with walking and passive commuting. A previous study, based on survey data of adults at work, showed that those who regularly commuted to work by bicycle had better mental health than those who walked to work, but no physical benefits were evident (20). In contrast, the present study found mental and physical health benefits of bicycling among adolescents. This finding suggests that bicycling to school is a more effective way to improve students’ health. The possible reason for this finding is that bicycling is more intensive than walking. Studies have shown that students whose home addresses are closer to school prefer to walk, and those who live a considerable distance away prefer to bicycle. When commuting times were similar between the two groups, students who commuted by bicycle traveled farther from home to school with the same amount of time. It meant that bicyclists did more exercise than those who commuted on foot (57). Although this study found more health benefits for bicycling than walking, we would like to emphasize that this does not negate the benefits of walking. This finding implies that, with regard to promoting students’ active commuting, the government should prioritize bicycling over walking. Additionally, we found that cycling can also prevent kidney disease, which has been confirmed in previous medical research (58). Active commuting has been shown to benefit health in studies combining walking and biking (6, 18, 24, 26, 59). However, as compared to other passive modes of transportation, bicycling was not associated with significant differences in adolescents’ health outcomes. This finding indicates that we should isolate the effects of bicycling trips from those of walking trips when studying active commuting among adolescents.

Finally, bicycling to school has health benefits for students living in rural and urban fringe areas, but not for those who live in urban centers. This finding was consistent with Lu (59), who conducted a cross-sectional study in Jiangsu, China, and found negative side effects of active commuting among urban residents than rural residents. One reason may be that with rapidly increasing motorization in China, traffic congestion is getting more serious, and those living in the city centers face more traffic and noise. The stress risks caused by a bad travel environment and air pollution may offset the potential positive effect of active commuting (60). We also found that bicycle commuting reduced the likelihood of sick leave absences among adolescents in rural areas. This finding may indicate that the benefits of bicycling to school are particularly beneficial for students living in rural areas.

## 5. Limitations

This study has limitations. First, we were only able to obtain relevant data up to the 2014–2015 wave, and changes in bicycle commuting and health output may have occurred since then. Scholars may continue to advance the research as new rounds of survey data become publicly available in the future. Second, due to data limitations, this study cannot cover detail of students’ commuting variables. Regarding commuting mode, we do not know the bicycling frequency and cannot observe whether there is a change in transportation mode, all of which may influence adolescents’ health. For example, a study by Ma et al. (16) investigated the relationship between cycling frequency and health, and found that only regular cycling reduced mental stress and increased life satisfaction, while cycling occasionally had no health-promoting effects found for those who cycled occasionally. Third, the health-related data in this study were derived from students’ self-reports, which may have some measurement bias compared to objective measurements. To improve the validity of the measures, future studies could collect objective data on student commutes and health from various perspectives. Fourth, a propensity score analysis can only partially overcome the omitted variable problem and cannot address reverse causality, and a longitudinal study is still needed to determine the causal association between bicycling and health outcomes. Finally, there may be other benefits of bicycle commuting, such as social dimensions, economic aspects, etc. that needs further discussion.

## 6. Implications

Adolescents’ health can be influenced by school travel mode, policymakers need to learn about this relationship to motivate and enhances adolescents’ wellbeing. First, this study shows that bicycling has more health benefits than walking. Based on this finding, the government should consider bicycling as an effective health promotion intervention than walking when it promotes active commuting among students. Policymakers also should deliver knowledge about the benefits of bicycling to parents and adolescents. Further, cities should improve bicyclists’ infrastructure, making active transport more appealing so that young people can experience those health benefits. Finally, we found that bicycle have a positive effect on improving the health of urban fringe and rural students. However, due to economic poverty, many families in rural areas cannot afford a bicycle. Thus, there should be adequate financial investments in rural areas in planning and management.

## 7. Conclusion

This study examines the effects of bicycling to school on various health outcomes among adolescents. Using national-level data from Chinese school-aged children (12–19 years of age). We found that bicycling to school has a positive effect on both subjective and physical health. Bicycling to school was associated with higher self-rated health, a healthier weight, and lower mental stress levels, as well as a lower risk of developing a brain disease. Additionally, we compared the bicycling group with both the walking and non-active travel groups separately. Compared with walking, cycling to school resulted in a higher self-rated health score and a lower mental stress score. Physically, students who bicycled to school were less likely to be absent from school and had kidney and brain diseases. However, we did not find a significant difference in health outcomes between cycling and other non-active modes of transportation.

## Data availability statement

The original contributions presented in the study are included in the article/supplementary material, further inquiries can be directed to the corresponding author.

## Ethics statement

Data collection was approved by the Ethics Committee of Renmin University of China, and each participant was informed of the purpose of this research. The participation of each participant in the study was voluntary, and they were assured that their privacy would be strictly protected.

## Author contributions

PD performed the statistical analysis and drafted the manuscript. CD critically revised and helped to draft the manuscript. SF helped perform the analysis with constructive discussions. All authors designed the protocol for this study, read, and approved the final manuscript.

## Funding

This research was funded by the National Natural Science Foundation of China (grant number 71871131) and the Fundamental Research Funds for the Central Universities (grant number CXJJ-2021-340).

## Conflict of interest

The authors declare that the research was conducted in the absence of any commercial or financial relationships that could be construed as a potential conflict of interest.

## Publisher’s note

All claims expressed in this article are solely those of the authors and do not necessarily represent those of their affiliated organizations, or those of the publisher, the editors and the reviewers. Any product that may be evaluated in this article, or claim that may be made by its manufacturer, is not guaranteed or endorsed by the publisher.

## Footnotes

^1^In China, elementary schools cover grades 1–6 (ages 6–12); middle schools cover grades 7–9 (ages 12–16). 99.68% of the participants in this study were students between the ages of 12 and 16.

^2^http://www.nhc.gov.cn/wjw/pqt/201803/a7962d1ac01647b9837110bfd2d69b26.shtml

^3^A person’s hukou is determined by the hukou of his or her parents and the place in which they reside. Agricultural and non-agricultural are the two types of hukou. There are different opportunities and restrictions associated with different hukou (35, 36).

^4^The parallel hypothesis is that there is no significant difference between the treatment and control groups in the matching variables; that is, the difference in the health outcomes between the two groups is entirely caused by the commute mode.
